# Natural history of conservatively managed nonfunctioning pituitary macroadenomas in the elderly: A study from the Swedish Pituitary Registry

**DOI:** 10.1007/s11102-026-01752-z

**Published:** 2026-08-20

**Authors:** Camilla Jerning, Charlotte Höybye, Sophie Bensing, Britt Edén Engström, Nasrin Al-Shamkhi, Bertil Ekman, Lorenza Bonelli, Henrik Borg, Pia Burman, Per Dahlqvist, Victor Hantelius, Oskar Ragnarsson, Anna Svensson, Jeanette Wahlberg, Anna-Karin Åkerman, Katarina Berinder, Maria Petersson

**Affiliations:** 1 Department of Molecular Medicine and Surgery, Karolinska Institutet and Department of Endocrinology, Karolinska University Hospital, Stockholm, Sweden; 2 Department of Medicine Solna, Karolinska Institutet and Department of Endocrinology, Karolinska University Hospital, Stockholm, Sweden; 3 Department of Medical Sciences, Endocrinology and Mineral Metabolism, Uppsala University and Uppsala University Hospital, Uppsala, Sweden; 4 Department of Endocrinology, Department of Health, Medicine and Caring Sciences, Linköping University, Linköping, Sweden; 5 Department of Endocrinology, Skåne University Hospital, Lund University, Malmö, Sweden; 6 Department of Endocrinology, Skåne University Hospital, Lund University, Lund, Sweden; 7 Department of Public Health and Clinical Medicine, Umeå University, Umeå, Sweden; 8 Department of Internal Medicine, School of Health and Medical Sciences, Örebro University, Örebro, Sweden; 9 Department of Internal Medicine and Clinical Nutrition, Institute of Medicine at Sahlgrenska Academy, University of Gothenburg, Gothenburg, Sweden

**Keywords:** Nonfunctioning pituitary macroadenoma, Adenoma size, Visual impairment, Hypopituitarism, Elderly patients, Conservative management

## Abstract

**Purpose:**

To investigate adenoma progression, pituitary function, visual impairment, and need for later surgery in elderly patients with conservatively managed nonfunctioning pituitary macroadenomas (macro-NFPAs).

**Methods:**

Patients ≥ 65 years in the Swedish Pituitary Registry diagnosed 1991–2023 with macro-NFPA and a decision for conservative management were included (*n* = 673, 38% females). Data at diagnosis and at 1- and 5-year follow-up, defined as assessments closest to 1 year (0.5–<2.5) and 5 years (2.5–<7.5), were retrieved. Surgery was recorded up to 7.5 years. Variables included adenoma size, pituitary function, visual impairment, and surgery. Patients were stratified into age groups 65–72, 73–80, and ≥ 81 years.

**Results:**

At diagnosis, mean age was 76 years, females were older (*p* < 0.001). Mean adenoma diameter and volume were 20 mm and 3067 mm³. Visual field defects and reduced visual acuity were seen in 20% and 12%, with no sex differences. Males had more hormone deficiencies (45% vs. 33%, *p* < 0.01). Hormone deficiencies and visual impairments increased with age (*p* < 0.001). The oldest subgroup had the largest adenomas (*p* < 0.01), with females having larger tumours (*p* < 0.05) and males more deficiencies (*p* < 0.05). Adenoma size, hormone deficiencies, and visual impairments remained stable over time within each age group. 6.7% underwent surgery during follow-up. In regression analyses, later surgery was associated with visual field defects at diagnosis (*p* < 0.05) but not with adenoma size.

**Conclusion:**

Pituitary surgery was uncommon in conservatively managed elderly patients with macro-NFPAs. Baseline visual field defects, but not tumour size, were associated with subsequent surgery, supporting regular individualised follow-up in these patients.

**Supplementary Information:**

The online version contains supplementary material available at https://doi.org/10.1007/s11102-026-01752-z.

## Introduction

Nonfunctioning pituitary adenomas (NFPAs) are often asymptomatic and when discovered incidentally, commonly referred to as pituitary incidentalomas. This also applies to macro-NFPAs (adenomas ≥ 10 mm), although these may affect surrounding structures and cause headaches, visual field defects, ophthalmoplegia, and hypopituitarism [1, 2]. The reported prevalence of NFPAs ranges from 7 to 41 per 100,000 individuals, with incidence rates generally below 3 per 100,000 person-years [2–4]. In Sweden, the incidence of macro-NFPA in the Swedish Pituitary Registry (SPR) has remained stable at approximately 2.0-2.2 reported cases per 100,000 since 2010 [5].

The incidence of diagnosed macro-NFPAs increases with age [6–8], and older adults (≥ 65 years) represent a growing proportion of cases, from approximately 15% in 2005 [9] to around 40% in recent SPR data [10].

Macro-NFPAs usually progress slowly [11], and current guidelines recommend multidisciplinary evaluation [12]. Macro-NFPAs that do not affect the optic chiasm are often managed conservatively, monitored with imaging, visual field examination and hormonal evaluation, whereas in cases with mass effect, transsphenoidal surgery (TSS) is the first-line treatment [11, 13]. Although TSS is generally considered safe, advancing age is associated with increased comorbidity, perioperative risk, and longer hospitalisation. Consequently, careful consideration of conservative management strategies is particularly important in elderly patients [7, 8, 14].

However, evidence guiding management in elderly patients is limited. Recommendations for follow-up regarding imaging, hormonal assessment, and visual evaluation have not been specifically validated in older individuals and are largely extrapolated from younger cohorts. Given the slow-growing nature of NFPAs, it remains unclear to what extent conservatively managed macro-NFPAs progress in elderly patients over time, or whether current monitoring strategies are clinically meaningful in this population. Data from the SPR provide a unique opportunity to study these outcomes in elderly patients.

The aim of this study was therefore to evaluate the natural course of macro-NFPAs over a five-year period in patients aged ≥ 65 years who were initially selected for conservative management and registered in the Swedish Pituitary Registry (SPR), focusing on adenoma progression, development of pituitary hormonal deficiencies, and visual impairments. Additionally, we investigated the proportion of patients who later underwent surgery following an initial conservative approach, with surgery recorded up to 7.5 years after diagnosis.

## Materials and methods

### The Swedish pituitary registry

The SPR, established in 1991, is a national quality registry that collects information on patients with pituitary adenomas, craniopharyngiomas, Rathke’s cleft cysts, and rarer pituitary diagnoses such as pituitary carcinomas. The registry is coordinated by a multidisciplinary team comprising endocrinologists, neurosurgeons, oncologists, pathologists, ophthalmologists, neuroradiologists, and endocrine nurses, representing all six healthcare regions in Sweden, and data are validated by local monitors. The data collected include diagnosis, age, sex, height, weight, radiological findings, results from ophthalmological examinations, hormonal evaluations, hormone replacement therapy, and working ability. Data on treatments including surgery, radiation and medical treatment are collected, as well as decisions from multidisciplinary team conferences (MDT), and data on treatment-related complications. For pituitary surgery, the registry has complete nationwide coverage, ensuring reliable capture of surgical interventions. All data are registered manually by treating physicians. Data on co-morbidities are not included in SPR.

The SPR records the presence or absence of pituitary hormone deficiencies as determined during routine clinical care. Pituitary function is assessed by the treating physician according to national clinical guidelines [15].

The SPR is integrated into the National Information Network for Cancer Treatment (INCA) IT-platform, hosted by the Regional Cancer Center (RCC) Stockholm-Gotland and receives financial support from the Swedish government.

### Study design

Patients aged 65 years or older at diagnosis who were diagnosed with a macro-NFPA between 1991 and 2023 with an initial decision for conservative management were requested from the SPR. To ensure inclusion of only patients with an initial conservative management strategy, 24 patients with unclear or absent documentation of initial management decision and who underwent pituitary surgery within the first year were excluded. These patients were included in the data extract from SPR despite a request for only conservatively managed cases. Available data did not indicate emergency indications or rapid tumour progression that would have prompted surgery, suggesting either delayed intervention or incomplete documentation.

Data on age, sex, adenoma size (volume and diameter), pituitary hormonal deficiencies, and visual impairment (visual field defects and impaired visual acuity) were collected at diagnosis and at follow-ups closest to 1 year (0.5 to < 2.5 years) and 5 years after diagnosis (2.5 to < 7.5 years). Imaging data were not centrally re-reviewed for this study but were assessed within routine clinical practice, often in multidisciplinary pituitary team conferences. Adenoma volume was calculated within the SPR from tumour width, height, and depth using the equation (width × height × depth)/2, as previously described [16].

Data on pituitary hormonal deficiencies were available for most patients, with missing data ranging from 2 to 5% for most variables and 13% for growth hormone deficiency.

Ophthalmological examinations are more commonly performed when chiasmal involvement is suspected. Consequently, visual data were missing or non-evaluable in approximately 20% of patients. Percentages of hormonal deficiencies and visual impairments are therefore reported as proportions of the total cohort, with data completeness for each variable specified as percentages in a footnote to Tables 1, 2 and 3.

“Any pituitary hormone deficiency” was defined as at least one deficiency in the pituitary hormonal axes assessed.

To assess potential era effects related to changes in imaging and clinical practice, a sensitivity analysis was performed comparing patients diagnosed in 1991–2009 with those diagnosed from 2010 onwards.

Information on surgical status (operated vs. non-operated) was retrieved for the entire follow-up period up to 7.5 years after diagnosis, meaning that surgery could occasionally be recorded after the five-year follow-up assessment.

To evaluate age-related differences, the cohort was stratified into three age groups: 65–72, 73–80, and ≥ 81 years.

### Ethics

This study and the Swedish Pituitary Registry were approved by the Ethics Committee at the Karolinska Institutet 2003 (Dnr 515/03) and 2012 (Dnr 915 − 32) supplemented 2024 (Dnr 2024-06273-02).

### Statistical methods

Data are presented as mean ± standard deviation (SD). Tumour volume data were log-transformed prior to analysis due to a non-normal distribution. Differences related to age, sex, and adenoma size (volume and diameter) were analysed with a two-way analysis of variance (ANOVA) or a mixed model ANOVA followed by Student’s t-test with Bonferroni correction, or a Student’s t-test only (age and tumour size total cohort). Hormonal deficiencies and visual impairments were evaluated with Chi-square test followed by Bonferroni correction when appropriate. P-values < 0.05 were considered statistically significant. Predictors of surgery were evaluated using multivariable logistic regression for outcomes during the follow-up period. Due to collinearity between adenoma diameter and volume, these variables were analysed in separate regression models. In the logistic regression analyses, missing data on visual field impairment and pituitary hormonal deficiencies were coded as absence of the condition, and patients with missing data on adenoma volume or adenoma diameter were excluded (complete-case analysis).

## Results

### Baseline characteristics of the macro-NFPA cohort at diagnosis

A total of 673 patients (258 females and 415 males) aged 65 years or older at the time of macro-NFPA diagnosis, with an initial decision for conservative management, were identified in the registry. Baseline data were similar across the six healthcare regions. The mean age was 76 ± 7 years, with females being older than males (77 vs. 75 years; *p* < 0.001). The mean adenoma diameter was 20 ± 6.7 mm, and the mean volume was 3067 ± 3896 mm^3^. There were no significant sex differences, although volume tended to be larger in females (*p* = 0.067).

No differences in age at diagnosis were observed between patients registered in the SPR before 2010 and those registered in 2010 and later. Mean tumour size and the prevalence of pituitary hormone deficiencies were slightly larger in patients registered before 2010 (diameter: 20.9 vs. 19.3 mm, 53% vs. 37%), likely reflecting early registry inclusion patterns. Importantly, diagnostic era did not influence the risk of subsequent surgery (Supplementary Table 1).

40% of the patients had at least one pituitary hormone deficiency at diagnosis, with a higher prevalence in males (*p* < 0.01). Gonadotropin deficiency was most common, followed by thyroid-stimulating hormone (TSH) and adrenocorticotropic hormone (ACTH) deficiencies, all being more frequent in males (*p* < 0.01). Antidiuretic hormone (AVP) deficiency was rare (*n* = 8) (Table 1).

**Table 1:** Clinical characteristics at diagnosis in patients aged ≥ 65 years with conservatively managed nonfunctioning pituitary macroadenoma

Clinical data	Total (*n* = 673)	Females (*n* = 258)	Males (*n* = 415)
Age (years)	76 ± 6.6	77 ± 7.2 ***	75 ± 6.1
Diameter (mm)	19.6 ± 6.7	20.1 ± 7.4	19.4 ± 6.1
Volume (mm^3^)	3067 ± 3896 (range: 180-41388)	3419 ± 4840 (range: 200-41388)	2848 ± 3158 (range: 180-36162)
Any pituitary deficiency	272 (40%)	84 (33%)	188 (45%) **
ACTH-deficiency^a^	117 (17%)	27 (10%)	90 (22%) **
GH-deficiency^a^	85 (13%)	27 (10%)	58 (14%)
LH/FSH-deficiency^a^	231 (34%)	65 (25%)	166 (40%) ***
TSH-deficiency^a^	156 (23%)	41 (16%)	115 (28%) **
AVP-deficiency	8 (1.2%)	3 (1.2%)	5 (1.2%)
Visual field defects^b^	134 (20%)	57 (22%)	77 (19%)
Visual acuity impairment^b^	82 (12%)	34 (13%)	48 (12%)

Data are shown as mean ± SD

Abbreviations: ACTH, adrenocorticotropic hormone; AVP, antidiuretic hormone; FSH, follicle-stimulating hormone; GH, growth hormone; LH, luteinizing hormone; TSH, thyroid-stimulating hormone

^a^ Number based on the total cohort. Missing or non-evaluable data were as follows: ACTH deficiency 3.1%, AVP deficiency 3.9%, GH deficiency 13%, LH/FSH deficiency 5.1%, and TSH deficiency 2.5%

^b^Number based on the total cohort. 537 (80%) of the patients were examined by an ophthalmologist

Statistical evaluation by Student´s t-test or chi-square test (categorical variables)

Tumour volume data were log-transformed prior to analysis due to non-normal distribution

** *p* < 0.01, *** *p* < 0.001 females vs. males

Patients with one or more hormonal deficiencies were older (77 ± 6.6 vs. 74 ± 6.2 years; *p* < 0.001) and had larger adenomas compared to patients with normal pituitary function (diameter: 21 ± 7.2 vs. 18 ± 5.4 mm, *p* < 0.001; volume: 3900 ± 4700 vs. 2108 ± 2369 mm^3^; *p* < 0.001).

Impaired visual fields and reduced visual acuity were reported in 20% and 12% of the patients, respectively, with no significant sex differences (Table 1).

Patients with visual impairments (*n* = 161) were older (79 ± 6.5 years vs. 75 ± 6.4; *p* < 0.001) and had larger adenomas (diameter: 22 ± 7.6 vs. 19 ± 6.1 mm, *p* < 0.001; volume: 4501 ± 5591 vs. 2615 ± 3051 mm^3^; *p* < 0.001).

### Stratification of the total cohort into age groups

There were approximately twice as many males as females in the two youngest age groups and mean age and adenoma size did not differ between sexes within these groups. Male patients in all three age groups more frequently had pituitary hormone deficiencies (*p* < 0.05), whereas the prevalence of visual impairments did not differ between males and females (Table 2).

**Table 2:** Clinical data at diagnosis in the different subgroups of patients aged ≥ 65 years with nonfunctioning pituitary macroadenoma managed conservatively

	Age: 65–72			Age: 73–80			Age: 81 and older		
	Total	Females	Males	Total	Females	Males	Total	Females	Males
Number	251	84	167	265	94	171	157	80	77
Age	69 ± 2.2	69 ± 2.2	69 ± 2.2	77 ± 2.3	77 ± 2.4	77 ± 2.2	85 ± 3.6	86 ± 4.1** (range: 81–105) *n* = 12 ≥ 90 years	84 ± 2.8 (range: 81–94) *n* = 4 ≥ 90 years
Diameter (mm)	18.1 ± 6.2	17.9 ± 5.7	18.3 ± 6.5	19.5 ± 6.0	18.8 ± 6.4	19.9 ± 5.8	22.2 ± 7.5	23.9 ± 8.7*^c, d^	20.5 ± 5.6^c^
Volume (mm^3^)	2533 ± 4038	2395 ± 4178	2602 ± 3977	2881 ± 2963	2687 ± 3717	2988 ± 2458	4252 ± 4752	5382 ± 6018**^c, d^	3078 ± 2443
Any pituitary deficiency^e^	82 (33%)	19 (23%)	63 (38%)*	108 (41%)	27 (29%)	81 (47%)*	83 (53%)	38 (48%)	45 (58%)
ACTH-def^a^	39 (16%)	8 (9.5%)	31 (19%)	41 (15%)	7 (7.4%)	34 (20%)*	37 (24%)	12 (15%)	25 (32%)*
GH-def^a^	24 (9.6%)	8 (9.5%)	16 (9.6%)	31 (12%)	6 (6.4%)	25 (15%)*	30 (19%)	13 (16%)	17 (22%)
LH/FSH-def^a^	70 (28%)	14 (17%)	56 (34%)**	91 (34%)	22 (23%)	69 (40%)*	70 (45%)	29 (36%)	41 (53%)*
TSH-def^a^	54 (22%)	14 (17%)	40 (24%)	57 (22%)	10 (11%)	47 (27%)*	45 (29%)	17 (21%)	28 (36%)*
AVP-def^a^	4 (1.6%)	2 (2.4%)	2 (1.2%)	3 (1.1%)	1 (1.1%)	2 (1.2%)	1 (0.6%)	0	1 (1.3%)
Visual field defects^b^	23 (9.2%)	7 (8.3%)	16 (9.6%)	63 (24%)^e^	23 (24%)	40 (23%)	48 (31%)^e^	27 (34%)	21 (27%)
Visual acuity impairment^b^	8 (3.2%)	3 (3.6%)	5 (3.0%)	32 (12%)^e^	12 (13%)	20 (12%)	42 (27%)^e^	19 (24%)	23 (30%)

Data are shown as mean ± SD

Abbreviations: ACTH, adrenocorticotropic hormone; AVP, antidiuretic hormone; FSH, follicle-stimulating hormone; GH, growth hormone; LH, luteinizing hormone; TSH, thyroid-stimulating hormone

^a^Number based on the total cohort. Missing or non-evaluable data were as follows: ACTH deficiency 3.1%, AVP deficiency 3.9%, GH deficiency 13%, LH/FSH deficiency 5.1%, and TSH deficiency 2.5%

^b^Number based on the total cohort. 537 (80%) of the patients were examined by an ophthalmologist

Statistical evaluation by a 2-way ANOVA followed by Student´s t-test with Bonferroni correction

Tumour volume data were log-transformed prior to analysis due to non-normal distribution

Age (between sex): F(1,657), *p* < 0.001

Diameter (between sex and age-groups): sex F(1,657) = 1.86, *p* = 0.17, age F(2,657) = 19.2, *p* < 0.001, interaction (sex/age) F(2,657) = 5.52, *p* = 0.0042

Volume (between sex and age-groups): sex F(1,657) = 0.38, *p* = 0.54, age F(2,657) = 22.9, *p* < 0.001, interaction (sex/age) F(2,657) = 5.43, *p* = 0.0046

For categorical variables, the chi-square test was used, followed by Bonferroni correction

^c^ Significantly different compared to age 65–72 years (males *p* < 0.05, females *p* < 0.01)

^d^ Significantly different compared to age 73–80 years (*p* < 0.01)

^e^ Significant increase with age *p* < 0.001

* *p* < 0.05, ** *p* < 0.01 females vs. males

In the oldest subgroup (≥ 81 years), the number of females and males was approximately equal, and females were slightly but significantly older at diagnosis than males (Table 2).

Females in the oldest age group had larger adenomas, both in diameter and volume, compared with males in the same age group (*p* < 0.05), and the adenomas were also larger compared with the females in both younger age groups (*p* < 0.01). The diameter of adenomas in males in the oldest group was also larger compared to the youngest group (65–72 years) (*p* < 0.05) (Table 2).

The likelihood of having at least one hormonal deficiency at diagnosis increased with age (*p* < 0.001). The prevalence of GH (*p* < 0.05) or gonadotropin (*p* < 0.01) deficiency also differed significantly between age groups. Visual field defects at diagnosis increased markedly with age, from 9.2% in the youngest group to 24% in the middle group and 31% in the oldest (*p* < 0.001). A similar age-related increase was observed for reduced visual acuity, with corresponding values of 3.2%, 12% and 27% (*p* < 0.001) (Table 2).

### Five-year follow-up

#### Characteristics of the five-year follow-up cohort

Among the total cohort, 628 patients did not undergo pituitary surgery during follow-up. Of these, 48% (*n* = 299) had available five-year follow-up data and constituted the five-year follow-up cohort. The median follow-up time of the five-year follow-up cohort was 55 months (IQR 41–64 months).

Of patients without five-year follow-up data, 93 patients were deceased, and 95 patients were diagnosed in 2020 or later and thus not yet eligible for five-year follow-up. The remaining patients were alive, but follow-up data within the time interval were missing.

To assess potential selection bias, patients without five-year follow-up data were compared with the five-year follow-up cohort. Patients without five-year data were on average one year older at diagnosis (*p* < 0.05) and had tumours with a slightly larger maximal diameter (20 ± 7.0 vs. 19 ± 6.3 mm; *p* < 0.05), but there were no differences in tumour volume, sex distribution, hypopituitarism, or visual impairment.

#### Changes over time in the patients who did not undergo surgery

**Fig. 1:**
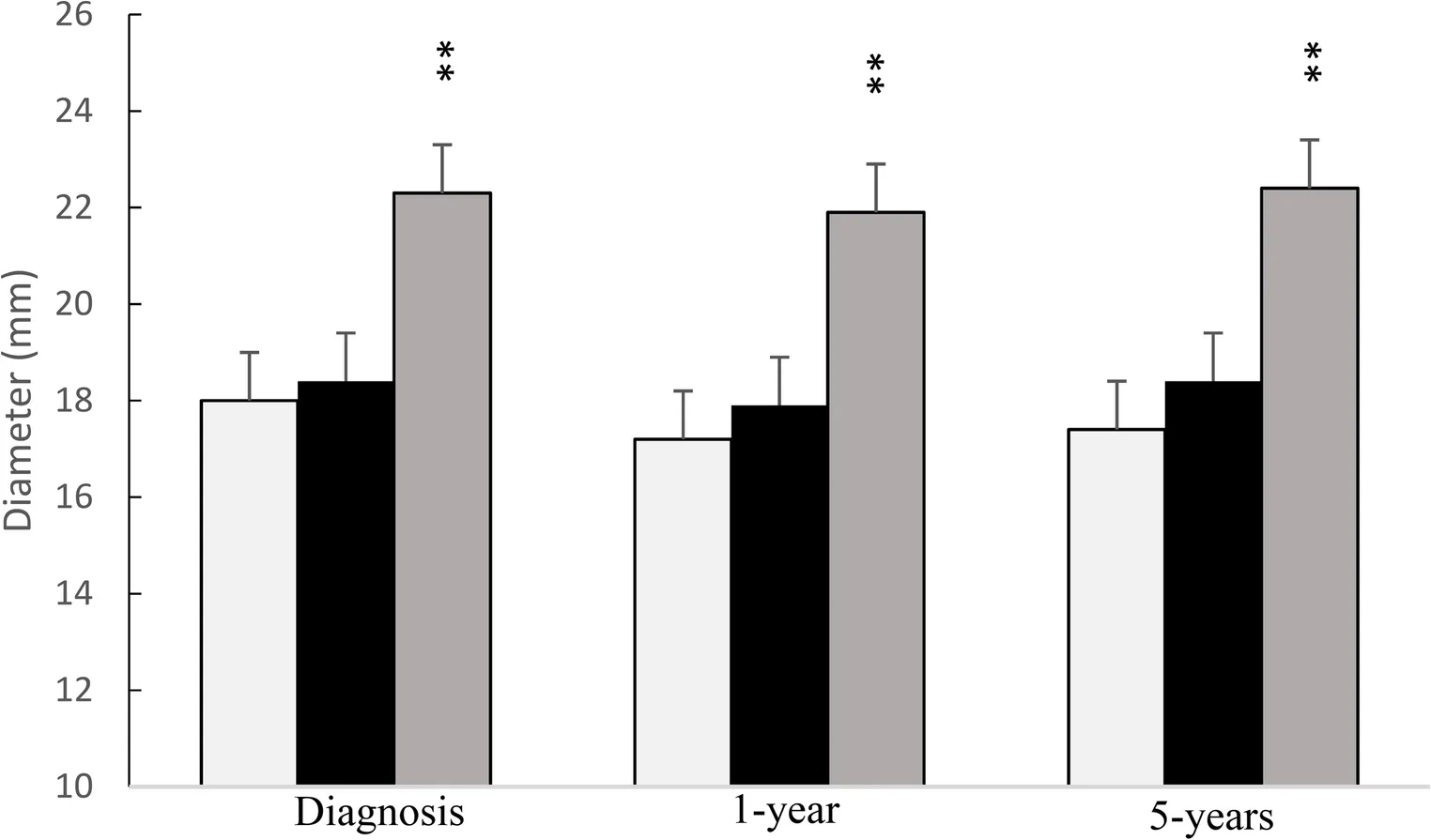
Diameter of the adenoma (mm) in the different age subgroups of the non-operated cohort at diagnosis, one year, and five years of follow-up. 65–72 years (white, *n* = 130), 73–80 years (black, *n* = 117), ≥ 81 years (grey, *n* = 52). Data are shown as mean ± SD. Statistical evaluation by a mixed model ANOVA followed by Student´s t-test with Bonferroni correction. Group F(2,748) = 19.45, *p* < 0.001, time F(2,748) = 0.83, *p* = 0.44, group x time F(4,748) = 0.99. ** *p* < 0.01 compared to both younger age groups

Mean adenoma size did not increase significantly over time (Fig. 1, Supplementary Table 2). This finding remained unchanged when restricting the analysis to patients with complete imaging data on adenoma size at diagnosis and at both follow-up time points (the diameter of the adenoma in this cohort was 18 ± 6.0 mm at diagnosis, 18 ± 6.7 mm at 1 year, and 18 ± 7.6 mm at 5 years (*n* = 183)).

At five-year follow-up, 45 patients (15%) in the non-operated cohort showed tumour growth of ≥ 3 mm in maximal diameter, and 71 patients (24%) demonstrated an increase in tumour volume of ≥ 20% compared with baseline. In contrast, maximal tumour diameter had decreased by ≥ 3 mm in 32 patients (11%), while 38 patients (13%) showed a reduction in tumour volume of ≥ 20% at the five-year follow-up.

The differences in volume and diameter between the oldest subgroup and the two younger groups persisted over time (Fig. 1, Supplementary Table 2).

At the cohort level, there was no significant increase in pituitary deficiencies over time. Among patients without pituitary hormone deficiencies at diagnosis, 19 (9.9%) had developed at least one new deficiency at the one-year follow-up and 29 (15%) at the five-year follow-up. Among patients with pituitary hormone deficiencies at diagnosis, 14 (13%) had developed additional deficiencies at one year and 23 (21%) at five years. Conversely, 12 (11%) and 16 (15%), respectively, no longer had any registered hormone deficiency at follow-up (Table 3).

**Table 3:** Changes in pituitary hormone deficiencies during follow-up in patients with and without deficiencies at diagnosis (*n* = 299)

	1-year follow-up	5-year follow-up
**No hormone deficiencies at diagnosis (***n* **= 191)**		
Developed ≥ 1 new deficiency compared to diagnosis	19 (9.9%)	29 (15%)
**≥1 hormone deficiency at diagnosis (n=108)**		
Developed additional deficiencies compared to diagnosis	14 (13%)	23 (21%)
No longer had any registered hormone deficiency	12 (11%)	16 (15%)

There was no significant increase in visual impairments over time, but the prevalence of visual field defects and visual acuity impairment was significantly higher in the 73-80-year group and the ≥ 81 group already at diagnosis. However, at the one- and five-year follow-ups, the differences in visual field defects were no longer statistically significant, whereas differences in visual acuity impairments persisted over time.

When analysing all age groups together, significantly more females compared to males had a visual acuity impairment at the one (*p* < 0.05) and five-year follow-up (*p* < 0.01). This difference was not significant when analysing the age groups separately (Supplementary Table 2).

### Patients who underwent surgery during follow-up

Out of the total cohort of 673 patients, 45 patients (6.7%) underwent pituitary surgery during the follow-up period (7.5 years) (13 females and 32 males, aged 65–82 years). The mean age of these patients at diagnosis was 71 ± 5.0 years, which was significantly younger compared to the non-operated group (75 ± 6.0 years; *p* < 0.001). Only 3 patients in the oldest subgroup (≥ 81 years) underwent surgery. There were no significant differences in age or adenoma size between females and males. The pattern of pituitary hormone deficiencies in females and males resembled that of the total cohort, although the differences were not statistically significant (Table 4).

**Table 4:** Clinical data at diagnosis and changes over time in patients who were initially managed conservatively but subsequently underwent surgery (*n* = 45). Patients were divided into two subgroups according to whether surgery had occurred by the five-year follow-up (defined as the assessment closest to 5 years after diagnosis, within 2.5–7.5 years). Eighteen patients had undergone surgery by the five-year follow-up, whereas 27 had not. As no significant sex differences were observed, data are presented combined

**At diagnosis**	All patients who later underwent surgery	Not operated by the five-year follow-up		Operated by the five-year follow-up
Number	45 (13 females)	27 (7 females)		18 (6 females)
Age	71 ± 5.0 (range: 65–82)	72 ± 5.1 (range: 65–82)		69 ± 4.5 (range: 65–80)
Time to surgery	3.4 years (range: 1.5-7 years)	3.8 years (range:1.5-7 years)^c^		2.8 years (range: 1.5-5 years)^c^
Diameter (mm)	19.4 ± 5.4	19.3 ± 6.1		19.6 ± 4.4
Volume (mm^3^)	2873 ± 2182	2865 ± 2494		2886 ± 1677
Any pituitary deficiency	18 (40%)	12 (44%)		6 (33%)
ACTH-def^a^	10 (22%)	7 (26%)		3 (17%)
GH-def^a^	2 (4.4%)	1 (3.7%)		1 (5.6%)
LH/FSH-def^a^	14 (31%)	8 (30%)		6 (33%)
TSH-def^a^	13 (29%)	10 (37%)		3 (17%)
AVP-def^a^	0	0		0
Visual field defects^b^	12 (27%)	7 (26%)		5 (28%)
Visual acuity impairment^b^	2 (4.4%)	1 (3.7%)		1 (5.6%)
**Changes over time**	Diagnosis		1-year	5-years
**Not operated by the five-year follow-up**	27 (7 females)		24 (7 females)^c^	24 (6 females)^c^
Adenoma size (mm/mm^3^)	19.3 ± 6.1 2865 ± 2494		20.5 ± 6.1 3571 ± 3238	22.6 ± 6.0* 5161 ± 5030*
Any pituitary deficiency	12 (44%)		12 (50%)	13 (54%)
Visual field defects^b^	7 (26%)		6 (25%)	14 (58%)*
Visual acuity impairment^b^	1 (3.7%)		2 (8.3%)	5 (21%)
**Operated by the five-year follow-up**	18 (6 females)		16 (4 females)^d^	18 (6 females)
Adenoma size (mm/mm^3^)	19.6 ± 4.4 2886 ± 1677		18.5 ± 4.8 2612 ± 1480	3.7 ± 4.7 83.6 ± 135
Any pituitary deficiency	6 (33%)		7 (44%)	8 (44%)
Visual field defects^b^	5 (28%)		4 (25%)	4 (22%)^Ɨ^
Visual acuity impairment^b^	1 (5.6%)		2 (13%)	0^Ɨ^

Data on age, sex, adenoma size (volume and diameter), pituitary hormonal deficiencies, and visual impairment (visual field defects and impaired visual acuity) were collected at diagnosis and at the follow-up closest to 1 year (0.5 to < 2.5 years) and closest to 5 years after diagnosis (2.5 to < 7.5 years)

Information on surgical status (operated vs. non-operated) was retrieved for the entire follow-up period up to 7.5 years after diagnosis, meaning that surgery could be recorded after the last available clinical follow-up data

Data are shown as mean ± SD

Abbreviations: ACTH, adrenocorticotropic hormone; AVP, antidiuretic hormone; FSH, follicle-stimulating hormone; GH, growth hormone; LH, luteinizing hormone; TSH, thyroid-stimulating hormone

^a^ Number based on the total cohort. Missing or non-evaluable data were as follows: ACTH deficiency 4.0%, AVP deficiency 2.2%, GH deficiency 13%, LH/FSH deficiency 2.2%, and TSH deficiency 2.2%

^b^ Number based on the total cohort. 35 (78%) of the patients were examined by an ophthalmologist

^c^ Data were missing for three patients at the one-year follow-up and for three other patients at the five-year follow-up; no patient had missing data at both follow-up assessments. One of the patients with missing five-year follow-up data underwent surgery 1.5 years after diagnosis and was therefore classified as non-operated during follow-up since no postoperative follow-up data were available

^d^ Data missing for 2 patients who had already undergone surgery

Statistical evaluation by a Mixed model ANOVA followed by Student´s t-test with Bonferroni correction

Tumour volume data were log-transformed prior to analysis due to non-normal distribution

Diameter: group F(1,107) = 25.1, *p* < 0.001, time F(2,107) = 5.43, *p* = 0.006, group x time F(2,107) = 28.29, *p* < 0.001

Volume: group F(1,107) = 51.5, *p* < 0.001, time F(2,107) = 25.5, *p* < 0.001, group x time F(2,107) = 76.9, *p* < 0.001

For categorical variables, the chi-square test was used, followed by Bonferroni correction

* *p* < 0.05, compared to diagnosis

Ɨ <0.05, compared to patients not operated on at the five-year follow-up

There were no significant differences between the operated and non-operated cohorts in the diameter or volume of the adenoma, the prevalence of pituitary hormone deficiencies or visual impairments at diagnosis or at the one-year follow-up (data for the non-operated cohort are presented in Supplementary Table 2).

In the total cohort, visual field defects at diagnosis were independently associated with subsequent surgery (OR 2.6–2.7, *p* < 0.05), whereas adenoma size and pituitary hormone deficiencies were not. Increasing age was associated with a lower likelihood of surgery (OR 0.85, *p* < 0.001) (Supplementary Table 3).

The average time from diagnosis to surgery was 3.4 years. By the time of the five-year follow-up, 18 patients had already undergone surgery, whereas 27 patients underwent surgery after the five-year follow-up but within the observation period of 7.5 years after diagnosis. These two groups did not differ significantly at diagnosis or at the one-year follow-up. However, among the patients who underwent surgery after the five-year follow-up, both adenoma size (*p* < 0.05) and the prevalence of visual field defects (*p* < 0.05) were increased at the five-year follow-up (Table 4).

Patients who had undergone surgery by the time of the follow-up assessments had fewer visual impairments after surgery compared with both their data at diagnosis (*p* < 0.05) and with the group of patients who underwent surgery after the last follow-up data collection (*p* < 0.05) (Table 4). The patients who were operated on after the follow-up assessments had a higher prevalence of visual field defects at the five-year follow-up compared with the large non-operated cohort (58% vs. 18%, *p* < 0.001; data for the non-operated cohort in the Supplementary Table 2).

## Discussion

In this nationwide cohort of patients aged ≥ 65 years with macro-NFPA and an initial decision for conservative management, 45 of 673 patients (6.7%) underwent surgical intervention during a follow-up period of up to 7.5 years. Overall, adenoma size, hormonal deficiencies, and visual impairments remained largely stable, and no cases of rapid tumour progression were observed. However, individual variation was seen both in pituitary function, with development of new deficiencies as well as resolution of previously recorded deficiencies, and in tumour size, with both growth and reduction observed over time. This heterogeneity in tumour behaviour supports the need for continued radiological monitoring or, at least, regular visual field assessment, even in clinically stable patients managed conservatively.

To our knowledge, no previous studies have specifically focused on elderly cohorts managed conservatively. In mixed-age cohorts of conservatively managed macro-NFPA, higher proportions of patients have been reported to require surgical intervention during follow-up, typically ranging between approximately 10–20% over comparable time periods [17–19]. Direct comparisons should, however, be interpreted with caution, as conservative management is less commonly adopted in younger patients, and tumour growth dynamics may differ with age. The low proportion of patients undergoing surgery in the present study should also be interpreted in the context of patient selection and competing risk factors. Elderly patients selected for conservative management may be frail, have a higher burden of comorbidity, increased mortality, and may be less likely to be able to tolerate surgical intervention, which may reduce the likelihood of surgery during follow-up. These factors have likely influenced both the initial MDT treatment decision and subsequent outcomes in the present study. In line with this, increasing age was independently associated with a lower likelihood of subsequent surgery in the multivariable logistic regression analysis.

This may be particularly evident in the oldest age group, where the very low proportion of patients undergoing surgery may reflect both an increasing comorbidity burden and the estimated time for clinically relevant tumour progression in older individuals.

However, as life expectancy increases, more elderly patients may be considered for surgery when clinically indicated, as studies have shown that TSS is generally well tolerated in this population [7, 14, 20, 21]. Nevertheless, morbidity, mortality, and length of hospitalisation increase with age in older patients undergoing TSS [22].

Notably, there were nearly twice as many males as females in the two youngest groups, possibly reflecting earlier detection of NFPA in premenopausal women [18]. Another contributing factor may be a higher frequency of imaging in men, who are overrepresented in emergency trauma units where neuroimaging is routinely performed [23]. A male predominance has also been observed in other studies [8, 14, 15, 24]. Despite this, adenoma sizes were similar in men and women, except in the oldest age group where females had larger adenomas. In contrast, males more frequently exhibited hormonal deficiencies, including ACTH and TSH deficiencies. The reasons for this remain unclear, but similar findings have been reported previously [15, 24]. Possible explanations include sex-specific differences in susceptibility to tumour-induced compression and ischaemia, as well as variations in tumour growth patterns [24–26].

Visual field impairment at baseline was associated with subsequent surgery in logistic regression analyses, reflecting that patients with visual impairments are more likely to be selected for surgical intervention at some point during follow-up. Consistent with baseline visual field impairment being associated with later surgery, patients who underwent subsequent surgery demonstrated an increase in visual field defects during follow-up, likely representing the indication for surgical intervention. Although postoperative outcomes were not the primary focus of this study, available data were included as part of the overall follow-up. Among operated patients with available follow-up data, postoperative improvement in visual outcomes was observed.

No patients received radiotherapy, likely reflecting patient selection, the absence of prior surgical intervention, and the delayed onset of radiotherapy effects, which may limit its use in elderly patients.

Despite the advanced age of the cohort, five-year follow-up data were available for nearly half of the patients. Missing follow-up was mainly due to death during the study period, or inclusion late in the study period without reaching the five-year time point. Other reasons for the lack of follow-up in the SPR may be that many stable patients, and especially older patients not expected to require surgery, are monitored at local centres or in primary care which do not report to the SPR. For comparison, in our previous study of patients with Rathke’s cleft cysts ≥ 10 mm (across all adult age groups), also based on the SPR, 42% had five-year follow-up [27], indicating that the follow-up coverage in the present study was relatively high given the older age of the cohort. In addition, the SPR has a high coverage rate of operated patients, as pituitary surgery is performed at tertiary centres reporting to the registry, supporting the accuracy of the low number of surgically treated patients in this study.

Although a majority of the patients underwent neuroophthalmological examination, some data were missing because examinations were not always performed in cases without chiasmal involvement. Visual field data should therefore be interpreted as descriptive registry data obtained during routine clinical care rather than as outcomes derived from a standardised study protocol. In addition, examination practices and diagnostic methods may have varied between centres and over the long study period.

Age-related eye diseases, such as glaucoma, diabetic retinopathy, or macular degeneration, may also have contributed to visual impairment in the oldest groups. Visual function nevertheless remained stable, with a non-significant decrease at five years, possibly reflecting registration bias or changes in examination methods, including increased use of optical coherence tomography (OCT) to assess chiasmal compression [28].

While TSS is generally safe and well-tolerated even in elderly patients [7, 14], surgical intervention should be avoided when not clinically necessary, particularly in the oldest age groups. In line with this, outcomes in the present study remained largely stable over time, with only a small proportion of patients undergoing surgery.

### Strengths and limitations

The main strength of this study is the use of a nationwide registry with long-term follow-up, allowing assessment of conservatively managed macro-NFPAs in an elderly population. The large cohort size and nationwide design provide a unique opportunity to evaluate long-term outcomes in elderly patients with macro-NFPA managed conservatively, a population that has been poorly studied previously.

Limitations include that follow-up data were not available for all patients, that visual impairment due to causes other than NFPA cannot be excluded, the lack of data on comorbidities, as such data are not registered in the SPR, and the absence of detailed information on the evaluation of pituitary function.

## Conclusion

The clinical course of conservatively managed macro-NFPAs in patients aged ≥ 65 years was largely stable over time, with minimal changes in adenoma size, pituitary deficiencies, or visual impairments. Only 6.7% of the entire cohort underwent later surgery, and no cases of rapid tumour progression were observed. Although interpretation of the very low surgical rate in the oldest age group should be made with caution, as age, frailty, comorbidity, and other clinical factors probably influenced treatment selection, subsequent surgery was uncommon in elderly patients initially selected for conservative management. These findings support continued monitoring with MRI, visual assessment, and pituitary function testing in this population.

## Supplementary Information

Below is the link to the electronic supplementary material.

Supplementary Material 1

Supplementary Material 2

Supplementary Material 3

## Data Availability

The data supporting the findings of this study are not publicly available due to legal and ethical restrictions related to patient confidentiality. The data originate from a Swedish national health register and can be accessed only upon reasonable request and with the necessary approvals from the responsible authorities.
