# Supplementary material for: Natural history of conservatively managed nonfunctioning pituitary macroadenomas in the elderly: A study from the Swedish Pituitary Registry
Source: Pituitary. 2026 Aug 20;29(5):147. doi: 10.1007/s11102-026-01752-z (PMC13493488; doi:10.1007/s11102-026-01752-z)
Supplement: Supplementary file 1 [file 11102_2026_1752_MOESM1_ESM.docx]

**Supplementary table 1**

**Clinical characteristics at diagnosis in conservatively managed patients aged ≥65 years with nonfunctioning pituitary macroadenomas: 1991–2009 vs 2010–2023.**

| **Variable** | **1991-2009** | **2010-2023** |
| --- | --- | --- |
| Number of patients | 154 | 519 |
| Age | 76±6.4 | 76±6.7 |
| Sex (females/males) | 50/104 | 208/311 |
| Diameter (mm) | 20.9±6.7** | 19.3±6.6 |
| Volume (mm^3^) | 3852±4752** | 2834±3574 |
| Any pituitary hormone deficiency | 82 (53%) | 190 (37%) |
| Visual impairments | 35 (23%) | 101 (19%) |
| Patients that underwent surgery | 9 (5.8%) | 36 (6.9%) |

Data are shown as mean ± SD. Statistical evaluation by Student´s t-test. ** p<0.01

Tumour volume data were log-transformed prior to analysis due to non-normal distribution.
