# Supplementary material for: Natural history of conservatively managed nonfunctioning pituitary macroadenomas in the elderly: A study from the Swedish Pituitary Registry
Source: Pituitary. 2026 Aug 20;29(5):147. doi: 10.1007/s11102-026-01752-z (PMC13493488; doi:10.1007/s11102-026-01752-z)
Supplement: Supplementary file 2 [file 11102_2026_1752_MOESM2_ESM.docx]

**Supplementary table 2**

**Clinical data at diagnosis and at one and five-year follow-up in the different subgroups of patients aged ≥65 years with nonfunctioning pituitary macroadenomas managed conservatively. Data of females and males are presented within parentheses.**

|  | **Total**  **(fem/male)**  **Mean age: 75±6.0**  **(76±6.6**  **/74±5.4)** |  |  | **Age 65-72**  **(fem/male)**  **Mean age: 69±2.1**  **(69±2.2/**  **69±2.0)** |  |  | **Age 73-80**  **(fem/male)**  **Mean age: 77±2.2**  **(77±2.2/**  **77±2.2)** |  |  | **Age 81 and older (fem/male)**  **Mean age: 84±2.7**  **(85±3.2***  **/83±1.5)** |  |  |
| --- | --- | --- | --- | --- | --- | --- | --- | --- | --- | --- | --- | --- |
| Time | Diagnosis | 1 year | 5 year | Diagnosis | 1 year | 5 year | Diagnosis | 1 year | 5 year | Diagnosis | 1 year | 5 year |
| Numbers. | 299 (114/185) | 280 (110/170) | 299 (114/185) | 130 (44/86) | 124 (43/81) | 130 (44/86) | 117 (40/77) | 110 (38/72) | 117 (40/77) | 52(30/22) | 46 (29/17) | 52(30/22) |
| Diameter (mm) | 18.9±6.3 (19.0±6.8 /18.9±6.0) | 18.2±7.3 (18.8±8.0/17.9±6.8) | 18.4±7.8 (17.9±7.6/18.6±7.2) | 18.0±6.3 (17.6±5.8/18.3±6.6) | 17.2±7.4 (17.5±7.8/17.1±7.3) | 17.4±7.7 (17.9±7.5/17.1±10.1) | 18.4±5.7 (17.7±6.8/18.8±5.0) | 17.9±6.7 (17.5±7.9/18.1±5.9) | 18.4±7.2 (16.0±6.0/19.5±7.5) | 22.3±6.8** (22.9±7.0/21.6±6.6) | 21.9±7.4 (23.0±7.2/20.2±7.4) | 22.4±8.9 (22.1±9.3/22.6±8.8) |
| Volume (mm^3^) | 2844±3871 (range: 180-32000)  (3131±4987 /2667±2984) | 2876±4762 (range: 0-50000)  (3467±6619/2485±2927) | 3178±6014 (range: 0-50000) (3065±6311/3241±5863) | 2433±4013 (2375±4826/2463±3551) | 2620±5607 (3154±8120/2321±3545) | 2698±5634 (3078±7795/2487±3998) | 2558±3180 (2545±4709/2566±1993) | 2628±3512 (2902±5211/2479±2138) | 2797±2936 (1865±1910/3226±3226) | 4534±4519** (5088±5243/3804±3315) | 4292±4823 (4906±5667/3217±2630) | 5278±7137 (5511±7172/5045±7365) |
| Any pituitary deficiency | 108 (36%) (28 (25%)/80 (43%))** | 101 (36%) (27 (25%)/74 (44%))** | 115 (38%) (31 (27%)/84 (45%))** | 39 (30%) (9 (20%)/30 (35%)) | 37 (30%) (10 (23%)/27 (33%)) | 38 (29%) (9 (20%)/29 (34%)) | 47 (40%) (8 (20%)/39 (51%))** | 41 (37%) (4 (11%)/37 (51%))*** | 48 (41%) (7 (18%)/41 (53%))*** | 22 (42%) (11 (37%)/11 (50%)) | 24 (52%) (12 (41%)/12 (71%)) | 26 (50%) (13 (43%)/13 (59%)) |
| ACTH-def^a^ | 49 (16%)(12 (11%)/37 (20%))* | 44 (16%) (13 (12%)/31 (18%)) | 54 (18%) (16 (14%)/38 (21%)) | 20 (15%) (5 (11%)/15 (17%)) | 18 (15%) (7 (16%)/11 (14%)) | 18 (14%) (6 (14%)/12 (14%)) | 17 (15%) (1 (2.5%)/16(21%))* | 14 (13%) (1 (2.6%)/13(18%)) | 23 (20%) (3 (7.5%)/20(26%))* | 12 (23%) (6 (20%)/6 (27%)) | 12 (26%) (5 (17%)/7 (41%)) | 13 (25%) (7 (23%)/6 (27%)) |
| GH-def^a^ | 28 (9.4%) (6 (5.3%)/22 (12%)) | 34 (12%) (6 (5.5%)/28(16%))* | 31 (10%) (8 (7.0%)/23(12%)) | 6 (4.6%) (1 (2.3%)/5 (5.8%)) | 14 (11%) (1 (2.3%)/13(16%))* | 14 (11%) (4 (9.1%)/10(12%)) | 15 (13%) (1 (2.5%)/14(18%))* | 13 (12%) (0/13 (18%))* | 12 (10%) (2 (5.0%)/10(13%)) | 7 (13%) (4 (13%)/3 (14%)) | 7 (15%) (5 (17%)/2 (12%)) | 5 (9.6%) (2 (6.7%)/3 (14%)) |
| LH/FSH-def^a^ | 92 (31%) (19 (17%)/73 (39%))** | 82 (29%) (13 (12%) /69 (41%))*** | 100 (33%) (21 (18%)/79 (43%))*** | 33 (25%)(7 (16%)/26 (30%)) | 28 (23%) (6 (14%)/22 (27%)) | 40 (31%) (11 (25%)/29 (34%)) | 41 (35%) (6 (15%) /35 (45%))* | 38 (35%) (2 (5.2%)/36 (50%))*** | 41 (35%) (5 (13%)/36 (47%))*** | 17 (32%) (6 (20%)/12 (55%))* | 16 (35%) (5 (17%)/11 (65%))** | 19 (37%) (5 (17%)/14 (64%))** |
| TSH-def^a^ | 62 (21%) (17 (15%)/45 (24%)) | 67 (24%) (19 (17%) /48 (28%))* | 81 (27%) (22 (19%)/59 (32%))* | 27 (21%) (9 (20%)/18 (21%)) | 24 (19%) (6 (14%)/18 (22%)) | 29 (22%) (9 (20%)/20 (23%)) | 23 (20%) (2 (5%)/  21 (27%))* | 26 (24%) (5 (13%)/21 (29%)) | 34 (29%) (3 (7.5%)/31(40%))*** | 12 (23%) (6 (20%)/6 (27%)) | 17 (37%) (8 (28%)/9 (53%)) | 18 (35%) (10 (33%)/8 (36%)) |
| AVP-def^a^ | 4 (1.3%) (1 (0.8%)/3 (1.6%)) | 5 (1.8%) (2 (1.8%)/3 (1.8%)) | 4 (1.3%) (1 (0.9%)/3 (1.6%)) | 2 (1.5%) (1 (2.3%)/1 (1.2%)) | 2 (1.6%) (1 (2.3%)/1 (1.2%)) | 1 (0.8%) (0/1 (1.2%)) | 1 (0.9%) (0/1 (1.3%)) | 1 (0.9%) (1 (2.6%)/0)) | 1 (0.9%) (1 (2.5%)/0)) | 1 (1.9%) (0/1 (4.5%)) | 2 (4.3%) (0/2 (12%)) | 2 (3.8%) (0/2 (9.1%)) |
| Visual field defects^b^ | 49 (16%) (19 (17%)/30 (16%)) | 42 (15%) (17 (15%)/25 (15%)) | 52 (18%) (18 (16%)/35 (19%)) | 8 (6.2%) (4 (9.1%)/4 (4.6%)) | 14 (11%)/(4 (9.3%) /10 (12%)) | 16 (12%) (5 (11%)/11 (13%)) | 27 (23%)** (9 (23%)/18 (23%)) | 17 (15%) (5 (13%)/12 (17%)) | 26 (22%) (6 (15%)/20 (26%)) | 14 (27%) ** (8 (27%)/6 (27%)) | 11 (24%) (8 (28%)/3 (18%)) | 11 (21%) (7 (23%)/4 (18%)) |
| Visual acuity impairment^b^ | 35 (12%) (15 (13%)/20 (11%)) | 30 (11%) (18 (16%)/12 (7.1%))* | 29 (9.7%) (17 (15%)/12 (6.5%))** | 5 (3.8%) (3 (6.8%)/2 (2.3%)) | 6 (4.8%)(3 (7.0%)/3 (3.7%)) | 3 (2.3%) (2 (4.5%)/1 (1.2%)) | 17 (15%)* (6 (15%)/11 (14%)) | 11 (10%) (4 (11%)/7 (9.7%)) | 17 (15%)** (8 (20%)/9 (12%)) | 13 (25%) *** (6 (27%)/7 (32%)) | 13 (28%) *** (11 (38%)/2 (12%)) | 9 (17%) ** (7 (23%)/2 (9.1%)) |

Abbreviations: ACTH, adrenocorticotropic hormone; AVP, antidiuretic hormone; FSH, follicle-stimulating hormone; GH, growth hormone; LH, luteinizing hormone; TSH, thyroid-stimulating hormone.

^a^ Number based on the total cohort. Missing or non-evaluable data were as follows: ACTH deficiency 2.0%, AVP deficiency 3.3%, GH deficiency 11%, LH/FSH deficiency 3.3%, and TSH deficiency 1.7%

^b^Number based on the total cohort. 245 (82%) of the patients were examined by an ophthalmologist.

Data are shown as mean ± SD. Statistical evaluation by a mixed model ANOVA or a 2-way ANOVA followed by Student´s t-test with Bonferroni correction.

Tumour volume data were log-transformed prior to analysis due to non-normal distribution.

Age: Sex F(1,293)=71.67, p<0.001

Diameter: Group F(2,748)=19.45, p<0.001, time F(2,748)=0.83, p=0.44, group x time F(4,748)=0.09, p=0.99

Volume: Group F(2,748)=18.27, p<0.001, time F(2,748)=1.18, p=0.31, group x time F(4,748)=0.13, p=1.0

For categorical variables, the chi-square test was used, followed by Bonferroni correction

* p<0.05, ** p<0.01, *** p<0.001 females vs. males or compared to the younger age groups (adenoma size and visual impairments)
