# Supplementary material for: Natural history of conservatively managed nonfunctioning pituitary macroadenomas in the elderly: A study from the Swedish Pituitary Registry
Source: Pituitary. 2026 Aug 20;29(5):147. doi: 10.1007/s11102-026-01752-z (PMC13493488; doi:10.1007/s11102-026-01752-z)
Supplement: Supplementary file 3 [file 11102_2026_1752_MOESM3_ESM.docx]

**Supplementary table 3**

**Multivariable logistic regression analyses of predictors of surgery**

Two separate multivariable logistic regression models were constructed because adenoma diameter and adenoma volume are highly correlated measures of adenoma size

3a.

| **Variable** | **Odds Ratio (OR)** | **95% CI** | **p-value** |
| --- | --- | --- | --- |
| Age (per year) | 0.85 | 0.80-0.91 | p<0.001 |
| Sex (male vs female) | 0.82 | 0.41-1.63 | p=0.56 |
| Adenoma volume at diagnosis | 1.66 | 0.73-3.77 | p=0.23 |
| Visual field impairment at diagnosis (yes vs no) | 2.68 | 1.25-5.74 | p=0.012 |
| Any pituitary hormone deficiency at diagnosis (yes vs no) | 1.25 | 0.64-2.43 | p=0.52 |

3b.

| **Variable** | **Odds Ratio (OR)** | **95% CI** | **p-value** |
| --- | --- | --- | --- |
| Age (per year) | 0.85 | 0.80-0.91 | p<0.001 |
| Sex (male vs female) | 0.81 | 0.41-1.63 | p=0.56 |
| Adenoma diameter at diagnosis | 1.01 | 0.96-1.06 | p=0.68 |
| Visual field impairment at diagnosis (yes vs no) | 2.59 | 1.20-5.55 | p=0.015 |
| Any pituitary hormone deficiency at diagnosis (yes vs no) | 1.21 | 0.62-2.36 | p=0.59 |

Complete-case analysis was performed. Ten patients were excluded from the multivariable analyses because of missing data on adenoma volume and/or adenoma diameter, leaving 663 patients for the regression analyses.

Adenoma volume was log-transformed prior to analysis.
